# Eating behavior, non-food substance consumption and negative urgency in women

**DOI:** 10.31744/einstein_journal/2020AO5269

**Published:** 2020-07-03

**Authors:** Jônatas de Oliveira, Táki Athanássios Cordás

**Affiliations:** 1 Hospital das Clínicas Faculdade de Medicina Universidade de São Paulo São PauloSP Brazil Programa de Transtornos Alimentares, Instituto de Psiquiatria, Hospital das Clínicas, Faculdade de Medicina, Universidade de São Paulo, São Paulo, SP, Brazil.

**Keywords:** Pica, Feeding behavior, Binge-eating disorder, Bulimia nervosa

## Abstract

**Objective:**

To evaluate aspects of eating behavior, presence of non-food substance consumption and negative urgency in women from an on-line support group for eating disorders.

**Methods:**

Participants (n=147) completed questionnaires for binge eating assessment, Intuitive Eating, negative urgency, cognitive restraint and a question of non-food substance consumption. Participants were separated according to criteria for bulimic symptoms and compulsive symptoms.

**Results:**

The consumption of non-food substances was 4.8% (n=7). The Bulimic Group (n=61) showed higher values for binge eating (p=0.01), cognitive restraint (p=0.01) and negative urgency (p=0.01) compared with the Compulsive Group (n=86). Only the Compulsive Group showed an inverse correlation between scores for binge eating and Intuitive Eating (p=0.01). In both groups, binge eating was inversely correlated with the subscale of body-food choice congruence of Intuitive Eating scale. As expected, the Bulimic Group reached higher values for measures of disordered behaviors such as cognitive restraint and binge eating, and lower scores for Intuitive Eating.

**Conclusion:**

The aspects of Intuitive Eating are inversely associated with compulsive and bulimic symptoms and the correlation analyses for binge eating and negative urgency agreed with models reported in published literature about negative urgency.

## INTRODUCTION

Eating behaviors and emotional regulation are severely altered by eating disorders (ED).^(1)^ Cases of binge eating disorders (BED) and bulimia nervosa (BN) are marked by changes in forms on how to deal with emotions, in addition to restrictive strategies for weight control that can increase enviroment vulnerability, and contribute to episodes of binge eating.^(2)^ Personality aspects contribute to episodes that can predict the occurrence of eating compulsions,^(1)^ specifically due to negative urgency (tendency to act impusilvily under negative influence) which has been related with severity of these behaviors and response to treatment.^(3)^ In the beginning of treatment patients with BED and high levels of negative urgency had lower benefit,^(3)^ in addition the reduction of negative urgency is also associated with recovery of ED.^(4)^

Considering the role of negative urgency and coping strategies in ED, a perspective little studied is the co-occurrence of Pica, an ED characterized by persistent consumption of non-food substance, which is not culturally accepted and considered inappropriate in the stage of development of individuals.^(5)^ Bryant-Waugh et al., published preliminary results of a new instrument for Pica,^(6)^ and, recently, findings have shown prevalence of diagnosis of Pica associated with ED in 1.3% (n=2 of 149) of patients, in addition to the consumption of non-food substances (plastic and gum) in outpatients who did not attend all criteria for Pica.^(7)^

## OBJECTIVE

To verify prevalence of self-reported consumption of non-food substances; to identify individuals with compulsive or bulimic behaviors, and to compare levels of negative urgency among groups.

## METHODS

This study was carried out in the Medical School of the *Universidade de São Paulo* (USP). Data collection was conducted using an on-line questionnaire applied to members of a Facebook^®^ private group for women who discuss the subject “living with ED”. No calculations were conducted, because questionnaires were made available to participants by the group administrators who were responsible to the page management. The study started in July 2018 and finished in August 2018. Those eligible for analysis were those who completed all steps of the questionnaire, and who had signed the study consent form of the Medical School of the *Universidade de São Paulo,* approval number 2.695.532, CAAE: 88846718.7.0000.0065.

### Exclusion criteria

We excluded from the study those younger than 18 years, foreigners, and those who reported to be pregnant or become pregnant within the last 6 months.

### Instruments

All instruments described in the following sections were transcribed to the on-line questionnaire, and the remaining information (age, weight, height, schooling, marital status) were self-reported in structured questions.

### Binge Eating Scale

Developed from the evaluation of presence and level of compulsive eating, the Binge Eating Scale (BES) has 62 affirmations in a Likert type scale that correspondent to the score from 0 to 3, which correspond from 0 up to 3 the maximal gravity of periodic binge eating (PBE), according to the authors.^(8)^Scores ranged from 0 to 64, and majority of the score indicated gravity of PBE. The scale also presents score of 17, and values from 0 to 17 for lack of compulsion, from 18 to 26 for moderate compulsion, and from 27 to 46 for severe binge eating. The scale was translated into Brazilian Portuguese in 2001,^(9)^ and the cohort score previous described was tested in a Brazilian study that compared with Brazilian version of the Structured Clinical Interview (SCID). Freitas et al., found sensibility of 97.9%, and test-retest reliability (after 15 days) estimated that Kappa coefficient was 0.65; weighted Kappa was 0.66. The Cronbach’s alpha found by authors were 0.89.^(10)^ In this study, we considered values >17 to select individuals with presence of PBE, and 18 to 26 for moderate level, and also were considered scores from 27 to 46 to describe individuals with severe PBE.

### Hay’s questionnaire

Consisted in structured questionnaire developed by Hay,^(11)^ which evaluated the frequency of BE behavior and inadequate compesantion methods in the last 3 moths. The answer’s option in this questionnarie are: “none”, “less than once per week”, “once a week”, and “two or more times per week”. The instrument was validated among adolecents (Kappa 0.50 for BE and 0.92 for self-induced vomiting).^(12)^ In this study, we considered only the answers “once a week” or “two or more times per week”, to select individuals with presence of inappropriate compensatory methods, because, according to Diagnostic and Statistical Manual of Mental Disorders, fifth edition (DSM-5), there is a need to presence of at least one compensatory episode in the last 3 months.^(5)^

### Subscale of negative urgency of Impulsive Behavior Scale

Whiteside et al., developed the Impulsive Behavior Scale (UPPS),^(13)^ which presented 45 questions in one Likert’s type scale of 4 score, which ranged from “strongly agree” to “strongly degree”. The instrument was adapated and validated for Brazilian Portuguese^(14)^ and posteriorly validated (α=0.85).^(15)^ In this study, we used only the instrument to the urgent subscale (12 questions), which evaluated tendency for experience impulses under negative affect condictions, and this was the largest scores related to higher urgency level.

### Cognitive Restraint Subscale (Three-Factor Eating Questionnaire)

Subscale of cognitive restraint measures the food restraint with the aim to modify the weight or body form, and it is part of the three-factor eating questionnaire, which, originally, has 21 items.^(16)^ According to description of Natacci et al.,^(17)^ the scale has the format of response from 4 score to items 1 to 20, and the numerical classification scale from 8 to question 21. In Brazil, we translated the scale into Portuguese in 2011^(17)^ and, posteriorly, we validated it by analysising convergent validation and discriminant, and also under standard of score for Brazilian population. Cronbach’s alpha described by authors were 0.83.^(18)^ In this study, we used only subscale of cognitive restraint, and this scores found was associated with large cognitive restraint.

### Investigation of non-food substance consumption

According to DSM-5 one of the criteria for the Pica diagnosis is the consumption of non-food substance within the last month.^(5)^ Considering the exploratory and non-diagnostics character of the study, we opted to investigate the occurrence of this behavior in the last 3 months to increase the chance of positive cases. To this, we created the following question: “Did you consume non-food substances (dust, brick, soap, paper or cotton), or any other substance that is not common food within the last 3 months?” (binary option of response: yes and no).

### Intuitive Eating Scale (version 2)

The original version of Intuitive Eating Scale (IES2) is composed by 23 scored items in Likert-type scale of 5 that ranges from 1 (strongly disagree) to 5 (strongly agree). This was adapted to the Brazilian Portuguese^(19)^ and applied to population with PEB, by presenting Cronbach’s alpha values of 0.61 to 0.74.^(20)^ The scale has four dimensions: “unconditional permission to eat” that reflects permission to eat food with no classification and restrictions for food groups; dimensions “eating for physical rather than emotional reasons” to investigate how choices are correlated with internal signs of eating and satiety; the dimension “reliance on hunger and satiety cues” to investigate the level of relaiance of individuals to the internal sings to guide their choices, and subscale “body-food choices congruence” to evaluate tendency of invididuals to choose in agreement with functioning and status of the body.^(21,22)^

### Criteria for group identification with compulsive symptoms and group with bulimia symptoms

According to DSM-5,^(5)^ we considered as criteria for bulimic symptoms the score >17 in BES characterizing the presence of binge eating^(9)^ and presence of purgation with frequency of, at least, once per week in the last 3 months, according to Hay’s questionnaire (we considered the options: “once a week” or “two or more times a week”). Criteria for binge symptoms were: score >17 in BES and have signed only the option “no time” for presence of purgation, according to Hay’s questionnaire.^(11)^

### Statistical analyses

Body mass index (BMI) was calculated according to form (weight/height^2^), and data were expressed in mean and standard deviation (SD) with measures of dispersion (minimal and maximum). To the comparison between groups, we conducted the non-paired t test using the Welch’s correction. We also conducted correlations (Spearman’s rank) between total score in BES with other means and also between scores for cognitive restraint with other measures. We considered a p<0.05 (GraphPad Prism 7.0).

## RESULTS

Considering the total evaluated (n=147), participants had on average 25 years of age (SD=5.44) ranging from 18 to 44 years. The BMI was 27.86Kg/m^2^ (SD=6.33) ranging from 17.9 to 50.57 (seven participants checked the option “I do not know my weight”). In BES, the total score was 30.81 (SD=7.28), with 31.3% (n=46) ranging from 18 to 26, and characterizing moderate binge eating and 68.7% (n=101) had score >26 (severe binge eating). In the IES2 the total score was 63.12 (SD=5.48), in the negative urgency subscale reached a mean of 38.01 (SD=6.26), and in cognitive restraint subscale the score was 16.16 (SD=4.19). According to criteria for bulimic symptoms (indications of BN) and BED, we identified 61 and of the 86 participants, respectively. In table 1, we schematized self-reported information of marital status, formal education, history of treatment for BN and BMI categories.

**Table 1:** General data of sample

Sample general data	Bulimic Group n (%)	Compulsive Group n (%)
Marital status		
Single	24 (39.3)	37 (43)
Married	13 (21.3)	27 (31.4)
Divorced	1 (1.6)	1 (1.2)
Dating	23 (37.7)	21 (24.4)
Formal education		
Primary education	-	1 (1.2)
Higher education (>15 year of formal education)	31 (50.8)	49 (57)
High education (11-14 year of formal education)	30 (49.2)	36 (41.0)
Underwent treatment for ED	31 (50.8)	45 (52.3)
Consumption of non-food substances	3 (3.5)	4 (6.6)
BMI categories		
Low weight	4 (7.1)	-
Eutrophy	19 (33.9)	27 (32.1)
Overweight	23 (41.1)	24 (28.6)
Obesity I	7 (12.5)	17 (20.2)
Obesity II and III	3 (5.4)	16 (19)

ED: eating disorders; BMI: body mass index.

### Consume of non-food substances

We found 7 women (4.8% compared to total) who reported non-food substance consumption within the last 3 months according to formulated issue. Characteristics of this groups were score in BES, on average, 34.57 (SD=7.76) (n=1 with <26 score, suggesting moderate binge eating, and n=6 with >26 score, suggesting severe binge eating). In these cases, four participants met criteria for Compulsive Group and three for the Bulimic Group. The BMI was, on average, 24.3kg/m^2^ (SD=5.15; minimal of 18 and maximal of 34.6). The total score for Intuitive Eating, the score was 60.57 (SD=7.36). In the subscale of negative urgency, the mean score was 38 (SD=7.83) and, in the cognitive restraint subscale, the score was 19.57 (SD=1.90).

### Comparison among groups

The Bulimic Group (n=61) answered the to score criteria >17 in BES characterizing presence of binge eating and presence of purgation with frequency of “once time/week” or “two or more times by per week”, according to the Hay’s questionnaire. This group was compared with participants who meet the indicative criteria of BED (>17 score in BES and have signed only the option “none” for presence of purgation). The Bulimic Group had greater values for BES (p>0.01), cognitive restraint (p=0.01), and urgency (p=0.02), in relation to Compulsive Group. In case of BMI and total scores in IES2 the Compulsive Group presented greater values (p=0.05 and 0.02, respectively) (Table 2).

**Table 2:** Comparison among groups

	Bulimic Group		Compulsive Group		p value	95%CI
	Medium (SD)	Minimal-maximum	Media (SD)	Minimal-maximum		
Age	25.03 (5.52)	18-44	25.6 (5.40)	18-38	0.5	-1.242-2.385
BMI	26.07 (5.55)	17.9-44.1	28.05 (6.53)	18.7-50.5	0.05	0.933-5.018
BES	33.54 (7.26)	18-46	28.87 (6.68)	18-44	<0.01	-6.999- -2.339
Cognitive restraint	17.23 (4.01)	6-24	15.41 (4.17)	7-23	0.01	-3.174- -0.4712
UPPS (negative urgency)	39.92 (6.03)	19-48	36.66 (6.13)	18-48	0.02	-5.264- -1.246
Intuitive Eating (total score)	61.44 (5.20)	48-70	64.31 (5.45)	52-76	0.02	1.122-4.621
UPE	14.98 (1.74)	11-21	14.36 (1.76)	10-18	0.03	-1.187- -0.0509
EAR	23.6 (2.17)	18-29	23.26 (2.52)	15-29	0.38	-1.11-0.432
RHC	12.66 (3.18)	5-19	14.69 (2.95)	5-20	0.01	1-3.05
BFC	4.13 (1.50)	2-8	4.87 (1.69)	2-8	0.01	0.216-1.27

Comparison conducted by non-pareated *t* test. BMI data in Bulimic Group (n=56) and Compulsive Group (n=84).

SD: standard deviation; 95% CI: confidence interval; BMI: body mass index; BES: binge eating scale: UPPS: Impulsive Behavior Scale; UPE: unconditional permission to eat; EAR: eating for physical rather than emotional reasons; RHC: reliance on hunger and satiety cues; BFC: Body-Food choice congruence.

The analysis in Bulimic Group showed positive correlation between BES and urgency (p<0.01) and subscale “eating for physical rather than emotional reasons” of the Intuitive Eating (p=0.01). We also observed inverse correlations between BES and subscales “Body-Food choice congruence” of Intuitive Eating (p=0.01) (Tables 3 and 4). In case of the Compulsive Group, we observed positive correlation between BES and negative urgency (p=0.01), and subscale “eating for physical rather than emotional reasons” of Intuitive Eating (p=0.02). The inverse correlations were found total score of Intuitive Eating (p=0.01) and subscales “reliance on hunger and satiety cues” (p=0.01) and “Body-Food choice congruence” (p=<0.01).

**Table 3:** Correlations with scores for Food Compulsion Scales

Correlations with BES	BMI	Cognitive restraint	UPPS (negative urgency)	Intuitive Eating (total score)	UPE	EAR	RHC	BFC
Bulimic Group, (n=61)	0.03 (0.80)	0.13 (0.31)	0.50 (<0.01)	-0.21 (0.11)	-0.14 (0.29)	0.32 (0.01)	-0.11 (0.37)	-0.38 (0.01)
Compulsive Group, (n=86)	0.15 (0.17)	-0.02 (0.78)	0.30 (0.01)	-0.36 (0.01)	0.19 (0.08)	0.24 (0.02)	-0.38 (0.01)	-0.45 (<0.01)

BMI data in Bulimic Group (n=56) and in Compulsive Group (n=84).

BES: binge eating scale; BMI: body mass index; UPPS: Impulsive Behavior Scale; UPE: unconditional permission to eat; EAR: eating for physical rather than emotional reasons; RHC: reliance on hunger and satiety cues; BFC: Body-Food choice congruence.

**Table 4:** Correlations with cognitive restraint

Correlation with cognitive restraint	BES	BMI	UPPS (negative urgency)	Intuitive eating (total score)	UPE	EAR	RHC	BFC
Bulimic Group, (n=61)	0.13 (0.31)	-0.47 (0.01)	-0.03 (0.78)	-0.26 (0.04)	-0.15 (0.25)	-0.30 (0.02)	-0.22 (0.09)	0.06 (0.61)
Compulsive Group, (n=86)	-0.29 (0.78)	-0.59 (<0.01)	-0.05 (0.95)	-0.06 (0.53)	-0.14 (0.21)	-0.37 (0.01)	0.02 (0.80)	0.12 (0.27)

BMI data in Bulimic Group (n=56) and in Compulsive Group (n=84).

BES: binge eating scale; BMI: body mass index; UPPS: Impulsive Behavior Scale; UPE: unconditional permission to eat; EAR: eating for physical rather than emotional reasons; RHC: reliance on hunger and satiety cues; BFC: Body-Food choice congruence.

## DISCUSSION

This study presents data of women who participated in an on-line support group in which they discussed topics related to ED. Of the sample, more than half of participants achieved score for severe binge eating. In both selected group, the majority of them were concentrated in eutrophic and overweight categories.

### Non-food substances consumption

In this study, we designed an exploratory question requesting response related to the last 3 months at the time of the research in order to increase the chance of positive reports. This criterion is different from the standard diagnostic criteria for Pica that consider the occurrence of ED behavior within the last month. Data indicated that 4.8% (n=7) had ED behavior. Of these, three cases of ingestion of non-food substance in the Bulimic Group and four cases in the Compulsive Group.

We highlight that, for diagnosis of Pica, there is need of other criteria and further investigation, that is, if the practice is culturally accept and, also, if the behavior occur in association with mental disorder such as intellectual deficiency, Autism Spectrum disorder or schizophrenia.^(5)^ In this study, we excluded participants who reported “current pregnancy or pregnancy within the last 6 months”, to avoid association with ingestion of non-food substances among this population.^(23)^

This was a non-diagnostic and exploratory approach study. There is need to highlight that craving for ingestion of non-food substances that can be characteristic of core ED, such as ice consumption (pagophagia) to suppress hunger, that, isolatedly, would not satisfy the Pica diagnosis.^(7)^

There is clinical challenge in characterization of Pica that, sometimes, is late, and may occur after negative consequences (intoxication, accidents, or admissions).^(24-26)^ Recently, a case of women from Cameroon (Africa) admitted to treat anemia and who reported intense wishing for comsumption of cauline (alumin silicate, using in the industry for fabrication of paper and poddling).^(25)^ The authors discussed the culture aspect determining the consumption (used for nausea relief), which avoided the diagnosis based on the DSM-5.^(5)^ In Mozambique, the consumption of mango with salt is common,^(27)^ however, in Brazil, there are publications that point out the comsuption of unripe mango with salt among pregnant women with Pica,^(28)^ although in east central region and northeast of the country, the mix (unripe mango with salt) is culturally accepted. Therefore, we emphasize the importance of considerer cultural aspects of regional eating and ingestion of any combination that already occurred before pregnancy, when it comes to study Pica among pregnant women.

Pregnant women with pagophagia (ice consumption) presented low consumption of carbohydrates, proteins, iron and zinc.^(29)^ A recent metanalysis confirmed the association of Pica with increased risk for low levels of hemoglobin, hematocrit, and plasmatic zinc.^(30)^ A qualitative study included a series of reports of pregnant women with Pica. Still, we showed that intense desire was the determinants for the consumption, such as in the following affirmation by one of the participants of the study: “I’m little reluctant if it is a very awkward thing, but it gets to a point that is difficult to avoid, the craving is so great, it makes your mouth water”.^(28)^Authors listed six determining categories for consumption of non-food substances such as “Motivation to practice”; “Personal history of practice”; “Desire satisfaction”; “Satisfaction in intake”; “Desire sharing”; “Family sharing”. Further studies in ED could benefit from use similar methodologies to depth the understandings of aspects and experiences behind the consumption of non-food substances.

### Eating Behavior

Tendency of episodes of binge eating under negative affect conditions is present characteristics in BN and BES, and attention and emotional conscience were identified as moderators of relations between compulsion and urgency.^(31)^ After control the effect of other variables, including impulsive aspects, Anestis et al., showed that negative urgency had relevant role in proposed model for BN.^(32)^ Negative urgency differed groups with participants with higher values for bulimic behaviors and also correlation with scores for binge eating in both situations.

During treatment with ED, a series of social skills are taught within behavioral cognitive therapy models and also dialectic behavior therapy that met characteristics of personality and specific psychopathology specific for each diagnosis.^(4)^ Manasse et al., showed that negative urgency functioned as limitation for treatment of 20 weeks.^(3)^ The contribution of authors in the study also applies to aspect of clinical decision making, because they develop skills to retain impulsive responses in context that negative affect is need for those with high level of urgency. Contribution of authors in study also applies to clinical decision making aspect, because they develop skills to retain impulsive answers in a context of negative affect that is necessary for those with high level of urgency. To identify previously individual with demands for specific therapies, such as dialectic behavior, can promote better adherence to treatments. In this study, seven women reported the consumption of non-food substances. Of these, six had score for severe binge eating. Still, in the analysis of correlation for both groups, scores of BES was positively correlated with urgent scores. Future studies must describe characteristics of personality and, perhaps, the presence of personality disorders associated with presence of BN or BED associated with Pica.

The Intuitive Eating has some facets such as “unconditional permission to eat” (no difference between groups) that propose the food intake associated with identification of physical need (hungry) and avoidance for food classification of food and food restraint; “eating for physical rather than emotional reasons” (no difference between groups) in which forms of treatment to understand emotional contexts included forms to understand emotional contexts that include food intake, avoiding the use emotional eating, and, in the end, “reliance on hunger and satiety cues” (higher in the Compulsive Group), which reflected how individuals respect and organize, according to intensity of internal feelings to start and finish a meal.^(21)^

The interoception awareness rescue is part of activities proposed by concept of Intuitive Eating, and according to study conducted in Brazil a number of studies have showed their relations with a healthy behavioral eating,^(33,34)^ and improvement after treatment.^(20)^ The analysis of correlation show inverse relation in Bulimic Group between binge eating and subscale “body-food choice congruence”.

The subscale “body-food choice congruence” evaluated how the individual adapts to food choices, considering the functioning and body status, and also how differentiate groups that presented higher values from the Compulsive Group. Both groups had inverse correlation between binge eating and the subscale “body-food choice congruence”.

We demonstrated that scores of Intuitive Eating were associated with low level of ED and positive body imaging.^(35)^ In addition, the Intuitive Eating is associated with lower practice of restrictive diets.^(35)^ Our data showed that cognitive restraint had negative correlation with BMI, both to the Bulimic Group, and to the Compulsive Group. However, in Bulimic Group eating restraint had inverse correlation with total score for Intuitive Eating, whereas the subscale “eating for physical rather than emotional reasons”, in both groups, had a negative correlation, given support to the opposition between constructs when the intuitive eating is considered as a healthy alternative for regulation of food behavior, in contrary to practice of restrictive diets.

### Limitations of the study

Data of this study was collected by self-reported instruments, mainly information such as weight, age and height of participations. Another limitation is the characteristic of non-diagnostic exploratory concerning the identification of consumption of non-food substances which must be carefully interpreted if compared with studies that present the diagnosis of Pica. We must consider that current results could not be extrapolated to population with ED due to lack of formal diagnosis to the compulsive and Bulimic Group.

## CONCLUSION

There is a co-occurrence of non-food substance intake associated with eating disorders behaviors. Most of studies conducted have presented a prevalence of Pica among pregnant women or in isolated cases, however, studies reporting the nosologic challenge of listing two different diagnoses such as Pica associated with bulimia nervosa or binge eating disorders. Future studies should consider qualitative approaches for groups with eating disorders in order to identify possible sociocultural factors that may determine these behaviors. The Bulimic Group had lower levels of Intuitive Eating and higher levels of urgency compared with the Compulsive Group. These findings confirmed the reports of previous models about the involvement of negative urgency in psychopathology of binge eating disorders and bulimia nervosa.
