# Supplementary material for: Eating behavior, non-food substance consumption and negative urgency in women
Source: Einstein (Sao Paulo). 2020 Jul 3;18:eAO5269. doi: 10.31744/einstein_journal/2020AO5269 (PMC7334001; doi:10.31744/einstein_journal/2020AO5269)
Supplement: Translation

# Translation: Comportamento alimentar, consumo de substâncias não alimentares e urgência negativa em mulheres

**Authors:** Jônatas de Oliveira, Táki Athanássios Cordás

**Affiliations:** 1 Brasil Programa de Transtornos Alimentares, Instituto de Psiquiatria, Hospital das Clínicas, Faculdade de Medicina, Universidade de São Paulo, São Paulo, SP, Brasil.

**Keywords:** Pica, Comportamento alimentar, Transtorno da compulsão alimentar, Bulimia nervosa

## Abstract

**Objetivo:**

Avaliar aspectos do comportamento alimentar, presença do consumo de substâncias não alimentares e urgência negativa em mulheres de um grupo de apoio para transtornos alimentares.

**Métodos:**

As participantes (n=147) preencheram instrumentos para avaliação de compulsão alimentar, Comer Intuitivo, urgência negativa, restrição cognitiva e uma questão para consumo de substâncias não alimentares. Elas foram divididas de acordo com critérios para sintomas bulímicos e sintomas compulsivos.

**Resultados:**

O consumo de substâncias não alimentares foi de 4,8% (n=7). O Grupo Bulímico (n=61) apresentou maiores valores para compulsão alimentar (p=0,01), restrição cognitiva (p=0,01), e urgência negativa (p=0,01) em relação ao Grupo Compulsivo (n=86). Apenas o Grupo Compulsivo demonstrou correlação inversa entre escores para compulsão alimentar e Comer Intuitivo (p=0,01). Para ambos os grupos, a compulsão alimentar foi inversamente correlacionada com a subescala de escolhas alimentares realizadas em congruência com o estado do corpo do Comer Intuitivo. Como esperado, o Grupo Bulímico apresentou maiores valores para medidas de comportamentos disfuncionais como restrição e compulsão alimentar, e menores escores para Comer Intuitivo.

**Conclusão:**

Os aspectos do Comer Intuitivo estiveram inversamente associados com os sintomas compulsivos e bulímicos, e os resultados das análises de correlação para Escala de Compulsão Alimentar e urgência negativa concordam com modelos da literatura sobre o envolvimento da urgência negativa.

## INTRODUÇÃO

Os comportamentos alimentares e a regulação emocional estão gravemente alterados nos transtornos alimentares (TA).^(1)^ Casos de transtorno da compulsão alimentar (TCA) e bulimia nervosa (BN) são marcados por alterações nas formas de lidar com emoções, além de estratégias restritivas para controle de peso, que podem aumentar a vulnerabilidade ao ambiente, contribuindo para os episódios de compulsão alimentar.^(2)^ Aspectos de personalidade contribuem e podem predizer a ocorrência de compulsões alimentares,^(1)^ especificamente a urgência negativa (tendência de agir impulsivamente sob afeto negativo) tem sido relacionada com a gravidade desses comportamentos e a resposta ao tratamento.^(3)^ Pacientes com TCA com altos níveis de urgência negativa no início do tratamento obtiveram menor benefício,^(3)^ e a diminuição da urgência negativa também está associada com a recuperação do TA.^(4)^

Considerando o papel da urgência negativa e das estratégias de enfrentamento das emoções nos TA, uma perspectiva pouco estudada é co-ocorrência com Pica, um TA caracterizado pelo consumo persistente de substâncias não alimentares, sendo não aceito culturalmente e inapropriado ao estágio de desenvolvimento do indivíduo.^(5)^ Bryant-Waugh et al., publicaram resultados preliminares de um novo instrumento para rastreamento de Pica,^(6)^ e, recentemente, foi demonstrada a prevalência de diagnóstico de Pica em associação com TA em 1,3% (n=2 de 149) dos pacientes, além do consumo de substâncias não alimentares (plástico e chiclete) em pacientes ambulatoriais que não atendiam a todos os critérios para Pica.^(7)^

## OBJETIVO

Verificar a prevalência do autorrelato de consumo de substâncias não alimentares; identificar indivíduos com comportamentos compulsivos ou comportamentos bulímicos; e comparar níveis de urgência negativa entre os grupos.

## MÉTODOS

Este estudo foi realizado na Faculdade de Medicina da Universidade de São Paulo (USP). A coleta de dados desta pesquisa foi conduzida por meio de um questionário on-line anônimo aplicado em participantes de um grupo fechado do Facebook^®^ exclusivo de mulheres que discutem a vivência dos TA. Não foram realizados cálculos de amostra, pois os questionários ficaram disponíveis para as participantes da página sob critério das administradoras do grupo de apoio, que fazem a gestão da página. Assim, a pesquisa foi iniciada em julho de 2018 com término em agosto de 2018. Foram elegíveis, para as análises, aquelas que completaram todas as etapas do questionário e que tinham assinalado no questionário a opção de concordância em participar do estudo de acordo com o Termo de Compromisso Livre e Esclarecido (TCLE) da Faculdade de Medicina da USP, número de aprovação 2.695.532, CAAE: 88846718.7.0000.0065.

### Critérios de exclusão

Foram desconsideradas do estudo as participantes menores de 18 anos, estrangeiras e as que assinalaram a opção gravidez atual ou nos últimos 6 meses.

### Instrumentos

Todos os instrumentos descritos nas próximas seções foram transcritos para o questionário on-line, e as informações restantes (idade, peso, altura, escolaridade e estado civil) foram autorrelatadas por questões estruturadas.

### Escala de Compulsão Alimentar

Desenvolvida para avaliar a presença e o nível de compulsão alimentar, a Escala de Compulsão Alimentar (BES - *Binge Eating Scale*) apresenta 62 afirmativas em uma escala do tipo Likert, que correspondem a um número de pontos de zero a 3, que vão desde a ausência (zero) até a gravidade máxima (3) da compulsão alimentar periódica (CAP), segundo os autores.^(8)^ Os escores variam de zero a 64 pontos, e maiores escores indicam gravidade da CAP. A escala também apresenta pontuação de corte de 17 pontos, sendo os valores de zero a 17 para ausência de compulsão; de 18 a 26, para compulsão moderada; e de 27 a 46 para compulsão alimentar severa. Foi traduzida para o português brasileiro em 2001,^(9)^ e o ponto de corte anteriormente descrito foi testado no Brasil em estudo que o comparou com versão brasileira da *Structured Clinical Interview* (SCID). Freitas et al., encontraram sensibilidade de 97,9%, e a confiabilidade teste-reteste (após 15 dias) estimada pelo coeficiente Kappa foi de 0,65; Kappa ponderado foi de 0,66. O alfa de Cronbach encontrado pelos autores foi de 0,89.^(10)^Neste estudo, foram considerados valores >17 para selecionar indivíduos com presença de CAP, sendo de 18 a 26 para nível moderado, e também foram consideradas as pontuações de 27 a 46 para descrever indivíduos com CAP grave.

### Questionário de Hay

Consiste em um questionário estruturado desenvolvido por Hay,^(11)^ que avalia a frequência de comportamentos de compulsão alimentar e os métodos compensatórios inapropriados nos últimos 3 meses. As opções de resposta neste questionário são: “nenhuma vez”, “menos que uma vez por semana”, “uma vez por semana” e “duas ou mais vezes por semana”. O instrumento foi validado em adolescentes (Kappa de 0,50 para compulsão alimentar e 0,92 para vômitos autoinduzidos).^(12)^ Nesse estudo, foram consideradas apenas as opções de resposta “uma vez por semana” ou “duas ou mais vezes por semana”, para selecionar indivíduos com presença de métodos compensatórios inapropriados, pois, segundo o Manual Diagnóstico e Estatístico de Transtornos Mentais – quinta edição (DSM-5), é necessária a presença de ao menos um episódio compensatório nos últimos 3 meses.^(5)^

### Subescala de urgência negativa da Escala de Comportamento Impulsivo

Whiteside et al., desenvolveram a Escala de Comportamento Impulsivo (UPPS),^(13)^ que apresenta 45 questões em uma escala do tipo Likert de 4 pontos, que varia de “concordo fortemente” a “discordo fortemente”. O instrumento foi adaptado e validado para o português brasileiro^(14)^ e posteriormente validado (α=0,85).^(15)^ Nesse estudo, foi utilizada apenas a subescala de urgência (12 questões), que avalia a tendência de experienciar impulsos sob condições de afeto negativo, sendo os maiores escores relacionados a um maior nível de urgência.

### Subescala de Restrição Cognitiva (*Three-Factor Eating Questionnaire*)

A subescala de restrição cognitiva afere a restrição alimentar com objetivo de modificar peso ou forma corporal e faz parte da *The Three-Factor Eating Questionnaire*, que, originalmente, possui 21 itens.^(16)^ Segundo a descrição de Natacci et al.,^(17)^ a escala possui formato de resposta de 4 pontos para os itens de 1 a 20, e uma escala de classificação numérica de 8 pontos para a questão 21. No Brasil, foi traduzida para o português em 2011^(17)^ e, posteriormente, foi validada com análise de validade convergente e discriminante, e também sobre padrões de pontuação para população brasileira. O alfa de Cronbach descrito pelos autores foi de 0,83.^(18)^ Nesse estudo, foi utilizada apenas a subescala de restrição cognitiva, sendo os escores encontrados associados com maior restrição cognitiva.

### Investigação de consumo de substâncias não alimentares

Segundo o DSM-5, para o diagnóstico de Pica, um dos critérios é o consumo de substâncias não alimentares no último mês.^(5)^ Considerando o caráter exploratório e não diagnóstico do estudo, optou-se por investigar a ocorrência deste comportamento nos últimos 3 meses para aumentar a chance de casos positivos. Para isso, foi formulada a seguinte questão: *“*Nos últimos 3 meses você apresentou comportamentos no qual consumiu substâncias não alimentares (terra, tijolo, sabonete, papel ou algodão) ou qualquer outra substância que não seja algo da alimentação normal?” (opção binária de resposta: sim e não).

### Escala de Comer Intuitivo (versão 2)

A versão original da Escala de Comer Intuitivo (*Intuitive Eating Scale –* IES2) é composta por 23 itens pontuados em uma escala do tipo Likert de 5 pontos, que varia entre 1 (“discordo fortemente”) até 5 (“concordo fortemente”). Foi adaptada para o português brasileiro^(19)^ e aplicada em população com CAP, apresentando valores de alfa de Cronbach de 0,61 a 0,74.^(20)^ A escala possui quatro dimensões: “Permissão incondicional para comer”, que reflete a permissão para comer alimentos, sem classificações e restrições para grupos alimentares; a dimensão “Comer por razões físicas ao invés de emocionais” investiga o quanto as escolhas estão atreladas com a sintonia aos sinais internos de fome e saciedade; a dimensão “Confiança nos sinais de fome e saciedade” investiga o nível de confiança que o indivíduo tem aos sinais internos para guiar suas escolhas; e a subescala “Escolhas alimentares realizadas em congruência com o estado do corpo” avalia a tendência do indivíduo para fazer escolhas de acordo com o funcionamento e o estado do corpo.^(21,22)^

### Critérios para identificação de grupo com sintomas compulsivos e grupo com sintomas bulímicos

De acordo com o DSM-5,^(5)^ foram considerados como critérios para sintomas bulímicos pontuação >17 na BES caracterizando presença de compulsão alimentar^(9)^ e presença de purgação com frequência de, ao menos, uma vez por semana nos últimos 3 meses, segundo o questionário de Hay (foram consideradas as opções: “uma vez por semana” ou “duas ou mais vezes por semana”). Os critérios para sintomas compulsivos foram: pontuação >17 na BES e ter assinalado apenas a opção “nenhuma vez” para presença de purgação, segundo o questionário de Hay.^(11)^

### Análises estatísticas

O índice de massa corporal (IMC) foi calculado segundo a fórmula (peso/altura^2^), e os dados foram expressos em média e desvio padrão (DP) com medidas de dispersão (mínimo e máximo). Para comparações entre grupos, foi realizado o teste *t* não pareado com correção de Welch’s. Foram também realizadas correlações (*r* de Spearman) entre escores totais na BES com outras medidas e também entre escores para restrição cognitiva com as outras medidas. Considerou-se p<0,05 (GraphPad Prism 7.0).

## RESULTADOS

### Características gerais da amostra

Considerando o total avaliado (n=147), as participantes tinham 25 anos em média (DP=5,44) com variação de 18 a 44 anos. O IMC foi de 27,86kg/m^2^ (DP=6,33) com variação de 17,9 a 50,57 (sete participantes assinalaram a opção “não sei o meu peso”). Na BES, a pontuação total foi 30,81 pontos (DP=7,28), com 31,3% (n=46) variando entre 18 a 26 pontos, caracterizando compulsão alimentar moderada e 68,7% (n=101) com pontuação >26 (compulsão alimentar grave). Na Escala de Comer Intuitivo a pontuação total foi 63,12 (DP=5,48); na subescala de urgência negativa atingiram 38,01 pontos em média (DP=6,26) e, na subescala de restrição cognitiva, 16,16 pontos (DP=4,19). Segundo os critérios para sintomas bulímicos (indicativos de BN) e compulsivos (indicativos de TCA), foram identificadas 61 e 86 participantes, respectivamente. Na tabela 1 foram esquematizadas as informações autorrelatadas de estado civil, escolaridade, histórico de tratamento para TA e as categorias de IMC.

**Tabela 1:** Dados gerais da amostra

Dados gerais da amostra	Grupo Bulímico n (%)	Grupo Compulsivo n (%)
Estado civil		
Solteira	24 (39,3)	37 (43)
Casada	13 (21,3)	27 (31,4)
Divorciada	1 (1,6)	1 (1,2)
Namorando	23 (37,7)	21 (24,4)
Escolaridade		
Ensino fundamental	-	1 (1,2)
Superior completo (>15 anos de educação)	31 (50,8)	49 (57)
Ensino médio (11-14 anos de educação)	30 (49,2)	36 (41,0)
Passou por tratamento para TA	31 (50,8)	45 (52,3)
Consumo de substâncias não-alimentares	3 (3,5)	4 (6,6)
IMC categorias		
Baixo peso	4 (7,1)	-
Eutrofia	19 (33,9)	27 (32,1)
Sobrepeso	23 (41,1)	24 (28,6)
Obesidade I	7 (12,5)	17 (20,2)
Obesidade II e III	3 (5,4)	16 (19)

TA: transtornos alimentares; IMC: índice de massa corporal.

### Consumo de substâncias não alimentares

Foram encontradas 7 mulheres (4,8% em relação ao total) que assinalaram consumo de substâncias não alimentares nos últimos 3 meses segundo a questão formulada. As características deste grupo foram pontuação na BES, em média, de 34,57 (DP=7,76) (n=1 com pontuação <26, indicando compulsão alimentar moderada, e n=6 com pontuação >26, indicando compulsão alimentar grave). Nestes casos, quatro participantes preencheram critérios para o Grupo Compulsivo e três para o Grupo Bulímico. O IMC foi, em média, 24,3kg/m^2^ (DP=5,15; mínimo de 18 e máximo de 34,6). O escore total para Comer Intuitivo foi de 60,57 pontos (DP=7,36). Na subescala de urgência negativa, a média foi de 38 pontos (DP=7,83) e, na subescala de restrição cognitiva, 19,57 pontos (DP=1,90).

### Comparações entre grupos

O Grupo Bulímico (n=61) atendeu aos critérios com pontuação >17 na BES caracterizando presença de compulsão alimentar e presença de purgação com frequência de “uma vez/semana” ou “duas ou mais vezes por semana”, segundo o questionário de Hay. Este grupo foi comparado com participantes que atenderam aos critérios indicativos de TCA (pontuação >17 na BES e ter assinalado apenas a opção “nenhuma vez” para presença de purgação). O Grupo Bulímico apresentou maiores valores para BES (p<0,01), restrição cognitiva (p=0,01) e urgência (p=0,02) em relação ao Grupo Compulsivo. No caso do IMC e escores totais na IES2, o Grupo Compulsivo apresentou valores maiores (p=0,05 e 0,02, respectivamente) (Tabela 2).

**Tabela 2:** Comparações entre grupos

	Grupo Bulímico		Grupo Compulsivo		Valor de p	IC95%
	Média (DP)	Mínimo-máximo	Média (DP)	Mínimo-máximo		
Idade	25,03 (5,52)	18-44	25,6 (5,40)	18-38	0,5	-1,242-2,385
IMC	26,07 (5,55)	17,9-44,1	28,05 (6,53)	18,7-50,5	0,05	0,933-5,018
BES	33,54 (7,26)	18-46	28,87 (6,68)	18-44	<0,01	-6,999- -2,339
Restrição cognitiva	17,23 (4,01)	6-24	15,41 (4,17)	7-23	0,01	-3,174- -0,4712
UPPS (urgência negativa)	39,92 (6,03)	19-48	36,66 (6,13)	18-48	0,02	-5,264- -1,246
Comer Intuitivo (escore total)	61,44 (5,20)	48-70	64,31 (5,45)	52-76	0,02	1,122-4,621
UPE	14,98 (1,74)	11-21	14,36 (1,76)	10-18	0,03	-1,187- -0,0509
EAR	23,6 (2,17)	18-29	23,26 (2,52)	15-29	0,38	-1,11-0,432
RHC	12,66 (3,18)	5-19	14,69 (2,95)	5-20	0,01	1-3,05
BFC	4,13 (1,50)	2-8	4,87 (1,69)	2-8	0,01	0,216-1,27

Comparações realizadas por meio do teste *t* não pareado. Dados do IMC no Grupo Bulímico (n= 56) e no Grupo Compulsivo (n= 84).

DP: desvio padrão; IC95%: Intervalo de confiança de 95%; IMC: índice de massa corporal; BES: Escala de Compulsão Alimentar; UPPS: Escala de Comportamento Impulsivo; UPE: permissão incondicional para comer; EAR: comer por razões físicas ao invés de emocionais; RHC: confiança nos sinais de fome e saciedade; BFC: escolhas alimentares realizadas em congruência com o estado do corpo.

As análises no Grupo Bulímico demonstraram correlação positiva entre a BES e urgência (p<0,01) e a subescala “Comer por razões físicas ao invés de emocionais” do Comer Intuitivo (p=0,01). Foram encontradas também correlações inversas entre BES e a subescala “Escolhas alimentares realizadas em congruência com o estado do corpo” do Comer Intuitivo (p=0,01) (Tabelas 3 e 4). No caso do Grupo Compulsivo, foi verificada correlação positiva entre a BES e urgência negativa (p=0,01) e a subescala “Comer por razões físicas ao invés de emocionais” do Comer Intuitivo (p=0,02). As correlações inversas foram encontradas para o escore total da Comer Intuitivo (p=0,01) e as subescalas “Confiança nos sinais de fome e saciedade” (p=0,01) e “Escolhas alimentares realizadas em congruência com o estado do corpo” (p=<0,01).

**Tabela 3:** Correlações com escores para a Escala de Compulsão Alimentar

Correlações com BES	IMC	Restrição cognitiva	UPPS (urgência negativa)	Comer Intuitivo (escore total)	UPE	EAR	RHC	BFC
Grupo Bulímico, (n=61)	0,03 (0,80)	0,13 (0,31)	0,50 (<0,01)	-0,21 (0,11)	-0,14 (0,29)	0,32 (0,01)	-0,11 (0,37)	-0,38 (0,01)
Grupo Compulsivo, (n=86)	0,15 (0,17)	-0,02 (0,78)	0,30 (0,01)	-0,36 (0,01)	0,19 (0,08)	0,24 (0,02)	-0,38 (0,01)	-0,45 (<0,01)

Dados do IMC no Grupo Bulímico (n=56) e no Grupo Compulsivo (n=84).BES: Escala de Compulsão Alimentar; IMC: índice de massa corporal; UPPS: Escala de Comportamento Impulsivo; UPE: permissão incondicional para comer; EAR: comer por razões físicas ao invés de emocionais; RHC: confiança nos sinais de fome e saciedade; BFC: escolhas alimentares realizadas em congruência com o estado do corpo.

**Tabela 4:** Correlações com restrição cognitiva

Correlações com restrição cognitiva	BES	IMC	UPPS (urgência negativa)	Comer Intuitivo (escore total)	UPE	EAR	RHC	BFC
Grupo Bulímico, (n=61)	0,13 (0,31)	-0,47 (0,01)	-0,03 (0,78)	-0,26 (0,04)	-0,15 (0,25)	-0,30 (0,02)	-0,22 (0,09)	0,06 (0,61)
Grupo Compulsivo, (n=86)	-0,29 (0,78)	-0,59 (<0,01)	-0,05 (0,95)	-0,06 (0,53)	-0,14 (0,21)	-0,37 (0,01)	0,02 (0,80)	0,12 (0,27)

Dados do IMC no Grupo Bulímico (n=56) e no Grupo Compulsivo (n=84).

BES: Escala de Compulsão Alimentar; IMC: índice de massa corporal; UPPS: Escala de Comportamento Impulsivo; UPE: permissão incondicional para comer; EAR: comer por razões físicas ao invés de emocionais; RHC: confiança nos sinais de fome e saciedade; BFC: escolhas alimentares realizadas em congruência com o estado do corpo.

## DISCUSSÃO

Este estudo apresentou dados de mulheres de um grupo on-line de apoio no qual se discutem temas relacionados ao TA. No total avaliado, mais da metade atingiu pontuação para compulsão alimentar grave. Em ambos os grupos selecionados, a maioria das participantes se concentrou nas categorias de eutrofia e sobrepeso.

### Consumo de substâncias não alimentares

Neste estudo, formulamos uma questão exploratória, incluindo os últimos 3 meses, para aumentar a chance de relatos positivos, diferente dos critérios diagnósticos para Pica, que consideram a ocorrência do comportamento no último mês. Os dados indicaram que 4,8% (n=7) apresentaram o comportamento, sendo três casos de ingestão de substâncias não alimentares no Grupo Bulímico e quatro casos no Grupo Compulsivo.

Destaca-se que, para o diagnóstico de Pica, são necessários outros critérios e investigações, como, por exemplo, se a prática é culturalmente aceita e, também, se o comportamento ocorre em associação a um transtorno mental como deficiência intelectual, Transtorno do Espectro Autista ou esquizofrenia.^(5)^ Neste estudo, foram excluídas as participantes que assinalaram a opção “gravidez atual ou nos últimos 6 meses”, para evitar a associação com o consumo de substâncias não alimentares prevalente nesta população.^(23)^ Este estudo teve caráter exploratório não diagnóstico, e também é necessário destacar que o consumo de substâncias não alimentares pode ser uma particularidade do TA nuclear, como, por exemplo, o consumo de gelo (pagofagia) para suprir a fome, que, isoladamente, não satisfaria um diagnóstico de Pica.^(7)^

Existe um desafio clínico na caracterização de Pica que, muitas vezes, é tardio, ocorrendo após as consequências negativas (intoxicação, acidentes ou internações).^(24-26)^ Recentemente, foi descrito o caso de uma mulher proveniente da República de Camarões (África) internada para tratamento de anemia que reportou desejos intensos para o consumo de caulino (silicato de alumínio, utilizado na indústria para fabricação de papel e porcelana).^(25)^ Os autores discutiam o aspecto cultural determinante do consumo (utilizado para alívio de náuseas), que impediu o diagnóstico segundo DSM-5.^(5)^ Em Moçambique, o consumo de manga com sal é comum,^(27)^ já, no Brasil, existem publicações que apontaram o consumo de manga verde com sal em gestantes como Pica,^(28)^ apesar de, na Região Centro-Oeste e Nordeste do país, a mistura ser culturalmente aceita. Assim, destaca-se a importância de considerar os aspectos culturais da alimentação local e se o consumo de alguma combinação já ocorria antes da gravidez, quando trata-se de uma investigação de Pica em gestantes.

Gestantes com pagofagia (consumo de gelo) apresentaram menor consumo de carboidratos, proteínas, ferro e zinco,^(29)^ e recente metanálise confirmou a associação de Pica com risco aumentado para baixos níveis de hemoglobina, hematócrito e zinco plasmático.^(30)^ Um estudo qualitativo abordou uma série de relatos de grávidas que reportaram Pica. Ainda, foi demonstrado que o desejo intenso era um dos determinantes de consumo, como no exemplo coletado pelos autores: “Eu fico relutando um pouco, se for uma coisa muito estranha, mas chega uma hora que fica difícil, porque a vontade é muito grande, você saliva muito”.^(28)^ Os autores listaram seis categorias determinantes para o consumo de substâncias não alimentares como “Motivação para a prática”; “História pessoal da prática”; “Realização do desejo”; “Satisfação na ingestão”; “Compartilhamento do desejo” e “Suporte familiar”. Futuros estudos na área de TA poderiam se beneficiar do uso de metodologias semelhantes, para aprofundamento dos aspectos e vivências por trás do consumo de substâncias não alimentares.

### Comportamento alimentar

A tendência de episódios de compulsão alimentar sob condições de afeto negativo é uma característica presente em BN e TCA, e a atenção e a consciência emocional foram identificadas como moderadoras da relação entre compulsão e urgência.^(31)^ Após controlarem o efeito de outras variáveis, inclusive de aspectos de impulsividade, Anestis et al., demonstraram que a urgência negativa teve papel relevante no modelo proposto para BN.^(32)^ A urgência negativa diferenciou os grupos com maiores valores para as participantes com presença de comportamentos bulímicos e também se correlacionou com escores para compulsão alimentar em ambos.

Durante o tratamentos dos TAs, uma série habilidades sociais são ensinadas dentro dos modelos de terapia cognitivo comportamental e também terapia comportamental dialética, que vão de encontro com as características de personalidade e psicopatologia específica de cada diagnóstico.^(4)^ Manasse et al., demonstraram que a urgência negativa atuou como limitadora para o tratamento de 20 semanas.^(3)^ A contribuição dos autores no estudo também se estende ao aspecto de tomadas de decisões clínicas, pois o desenvolvimento de habilidades para reter respostas impulsivas no contexto de afeto negativo é necessário para aqueles com alto nível de urgência. Identificar previamente indivíduos com demandas de terapias específicas, como a comportamental dialética, pode promover melhor aderência aos tratamentos.

Neste estudo, sete mulheres reportaram o consumo de substâncias não alimentares e, destas, seis apresentaram pontuação para compulsão alimentar grave. Ainda, nas análises de correlação para ambos os grupos, os escores da BES se correlacionaram positivamente com escores de urgência. Futuros estudos devem descrever características de personalidade e, talvez, a presença de transtornos de personalidade associados com a presença de BN ou TCA associado com Pica.

O Comer Intuitivo possui algumas facetas como a “Permissão incondicional para comer” (sem diferença entre grupos), que propõe o consumo alimentar associado com a identificação de necessidade física (fome) e a evitação para classificações de alimentos e restrição alimentar; “Comer por razões físicas ao invés de emocionais” (sem diferença entre grupos), que trata de formas de entender os contextos emocionais que permeiam o consumo alimentar, evitando o uso do comer emocional; e, por fim, a “Confiança nos sinais de fome e saciedade” (maior no Grupo Compulsivo), que reflete quanto os indivíduos respeitam e se organizam, de acordo com a intensidade das sensações internas para iniciar e terminar a refeição.^(21)^

O resgate da consciência interoceptiva faz parte das atividades propostas pelo conceito do Comer Intuitivo, e diversos estudos demonstram sua relação com comportamentos alimentares saudáveis^(33,34)^ e melhora após tratamento, segundo estudo realizado no Brasil.^(20)^ As análises de correlação demonstraram relação inversa no Grupo Bulímico entre compulsão alimentar e a subescala “Escolhas alimentares realizadas em congruência com o estado do corpo”, enquanto no Grupo Compulsivo a compulsão alimentar apresentou relação inversa com o escore total da IES2 as subescalas “Confiança nos sinais de fome e saciedade” e “Escolhas alimentares realizadas em congruência com o estado do corpo”.

A subescala “Escolhas alimentares realizadas em congruência com o estado do corpo” avalia o quanto o indivíduo adapta escolhas alimentares, considerando o funcionamento e o estado do corpo, e também diferenciou os grupos apresentando maiores valores para o Grupo Compulsivo. Ambos os grupos apresentaram correlação inversa entre compulsão alimentar e a subescala “Escolhas alimentares realizadas em congruência com o estado do corpo”.

Foi demonstrado que escores do Comer Intuitivo foram associados com menor nível de comer transtornado e com imagem corporal positiva.^(35)^ Além disso, o Comer Intuitivo está associado com menor prática de dietas restritivas.^(35)^ Nossos dados demonstraram que restrição cognitiva apresentou correlação negativa com IMC, tanto para o Grupo Bulímico, como no Grupo Compulsivo. Apenas no Grupo Bulímico, a restrição alimentar apresentou correlação inversa com a pontuação total para Comer Intuitivo, enquanto para a subescala “Comer por razões físicas ao invés de emocionais”, em ambos os grupos, a correlação foi negativa, suportando a oposição entre construtos quando considera-se o Comer Intuitivo como alternativa saudável para regulação do comportamento alimentar, ao invés da prática de dietas restritivas.

### Limitações do estudo

As informações deste estudo foram coletadas por meio de instrumentos de autorrelato, principalmente dados de peso, idade e altura. Destacamos também o caráter exploratório não diagnóstico relacionado com a questão que identifica consumo de substâncias não alimentares, que deve ser interpretado com cautela, quando comparado aos estudos que apresentam o diagnóstico de Pica. Também deve-se considerar que os presentes resultados não podem ser extrapolados para populações com TA, devido à falta de um diagnóstico formal, no que se refere aos Grupos Compulsivo e Bulímico.

## CONCLUSÃO

Existe co-ocorrência de consumo de substâncias não alimentares em associação com comportamentos alimentares transtornados. A maioria dos estudos já realizados apresentou prevalência de Pica em gestantes ou em casos isolados, sendo necessários estudos que relatem o desafio nosológico de listar dois diagnósticos distintos como Pica associados com bulimia nervosa ou transtorno da compulsão alimentar. Destaca-se a necessidade do uso de abordagens qualitativas em futuros estudos em grupos com transtornos alimentares para identificação dos possíveis determinantes socioculturais destes comportamentos. O Grupo Bulímico apresentou menores níveis de Comer Intuitivo e maiores níveis de urgência em relação ao Grupo Compulsivo, confirmando modelos anteriores sobre o envolvimento da urgência negativa na psicopatologia de transtorno da compulsão alimentar e bulimia nervosa.
